# Preliminary quantitative myocardial perfusion in response to cold pressor test in normals

**DOI:** 10.1186/1532-429X-15-S1-E49

**Published:** 2013-01-30

**Authors:** Yi Wang, Mike Passic

**Affiliations:** 1 St. Francis Hospital, Roslyn, NY, USA

## Background

Echo and nuclear studies have shown that coronary flow changes with the cold pressor test (CPT). CPT has been used to measure coronary flow response because it provides an endothelium-dependent coronary vasodilation that is sensitive for detecting early changes in coronary endothelial function. There was also a study showed myocardial perfusion increases during CPT with first pass perfusion imaging [1]. We studied the regional myocardial perfusion values in normal volunteers under stress (CPT), at rest, and myocardial flow reserve (MFR).

## Methods

To ensure the normality of the data, strict exclusion criteria were used in volunteer recruitment, including exclusion of hypertension, diabetes, smoking, family history of cardiac disease, a cardiac ultrasound and CT coronary calcium score ≤20. Three volunteers (ages: 38.3±9.8, 1 female) were enrolled after IRB approval. All subjects were injected with a 0.05 mM/kg Gd at 6 ml/s, with additional 0.005 mM/kg Gd injection immediately prior to it (dual bolus injection). During the contrast administration, the first pass perfusion imaging under CPT stress was performed on a 1.5T scanner (Siemens, Malvern, PA) by putting the right hand in an icy water bath for 90 seconds before the perfusion imaging. A saturation recovery SSFP technique was used for both CPT and the 30-minute later resting perfusion. A voxel spatial resolution of 1.9×2.8×8 mm^3^ was achieved in 3 rotational long axis slices per heartbeat over 50 heartbeats. Mean signal intensities of all pixels in each of the six myocardial segment at every time point were transferred to custom developed program to calculate absolute regional myocardial flow. To solve the deconvolution equation, a Fermi function was selected as the distribution of tracer residence times to search for the best fit of the myocardial dynamic signal curve for each sector, with LV blood signal as arterial input function. A total of 18 segments per volunteer were evaluated. The MFR was determined as the ratio of stress to rest perfusion.

## Results

All volunteers were able to keep the hand in the ice-water bath for the whole CPT. The mean and standard deviation of rest perfusion for all volunteers were 0.55 ± 0.27 ml/g/min, 0.57 ± 0.18 ml/g/min during CPT, and 1.15 ± 0.23 of MFR, respectively, n = 54. The mean perfusion between CTP and rest was not significant.

## Conclusions

Absolute quantification of myocardial blood flow with dual bolus injection during CPT is feasible. Contrary to previous study [1], mean perfusion during CPT showed a moderate increase compare to rest, but not significant in Normals. Further studies with Normals and patients with known cardiac risk factors are needed to validate these findings.

## Funding

St. Francis Research Foundation.
